# Gender perspective in medicine: a vital part of medical scientific rationality. A useful model for comprehending structures and hierarchies within medical science

**DOI:** 10.1186/1741-7015-4-20

**Published:** 2006-08-24

**Authors:** Gunilla Risberg, Katarina Hamberg, Eva E Johansson

**Affiliations:** 1 Department of Public Health and Clinical Medicine, Section of Family Medicine, Umeå University, NUS, S-90185 Umeå, Sweden

## Abstract

**Background:**

During the past few decades, research has reported gender bias in various areas of clinical and academic medicine. To prevent such bias, a gender perspective in medicine has been requested, but difficulties and resistance have been reported from implementation attempts. Our study aimed at analysing this resistance in relation to what is considered good medical research.

**Method:**

We used a theoretical model, based on scientific competition, to understand the structures of scientific medicine and how they might influence the resistance to a gender perspective in medicine. The model was originally introduced to discuss how pluralism improves rationality in the social sciences.

**Results:**

The model provided a way to conceptualise different fields of research in medicine: basic research, applied research, medical philosophy, and 'empowering' research. It clarified how various research approaches within medicine relate to each other, and how they differ and compete. It also indicated why there might be conflicts between them: basic and applied research performed within the biomedical framework have higher status than gender research and other research approaches that are performed within divergent research paradigms.

**Conclusion:**

This hierarchy within medical research contributes to the resistance to a gender perspective, causing gender bias and making medical scientific rationality suboptimal. We recommend that the theoretical model can be applied in a wider medical context when different and hierarchically arranged research traditions are in conflict. In this way, the model might contribute to shape a medical community where scientific pluralism is acknowledged to enlarge, not to disturb, the scientific rationality of medicine.

## Background

The concept of gender has been used in the social and humanistic sciences since the 1960s. It was originally introduced to designate how different societies and cultures interpret biological sex [1]. It refers to the constantly ongoing social construction of what is considered 'feminine' and 'masculine' ('doing gender'), based on power and sociocultural norms about women and men [2-4].

Thus gender is a wider concept than sex, [3] and a gender perspective in medicine implies that living conditions, positions in society and societal expectations about 'femininity' and 'masculinity' should be considered along with biology in professional relationships, as well as when theorising about women and men. Unawareness of gender aspects among health-care professionals and medical researchers can lead to gender bias.

Such bias has been described during the past decades in research from various areas of clinical and academic medicine. Unjustified differences in the investigation and treatment of male and female patients have been identified, most abundantly and ever recurrently in coronary heart disease [5-7], but also in other conditions such as kidney disease [5], depression [8], HIV/AIDS [9], colorectal cancer [10], neck pain [11] and irritable bowel syndrome[12]. There are also reports of discrimination and harassment based on gender among medical students [13], physicians [14], medical faculty [14,15] and medical researchers [15,16].

To prevent and avoid gender bias, there are good reasons for a gender perspective to be included in medicine in the same way as perspectives regarding social class, ethnicity, and age. However, in reports from attempts to introduce gender issues in clinical medicine, medical research and medical curricula, difficulties and resistance have been experienced and described. For example, in medical research and education, the term 'gender' is often wrongly used as being synonymous with biological sex [17]. Sometimes 'sex' is simply replaced by 'gender', and used as a variable even in experiments on animals [18]. Analyses of medical textbooks, training material and examination questions have revealed stereotypical gender patterns and even open patriarchal views [15,19-21]. Enduring and difficult work has been described when trying to address and change this [20-23]. Lack of interest in gender issues has been shown from students [23,24] as well as from faculty. Interviews with course organisers at a medical school revealed that several of them found a gender perspective to be of little relevance in medicine; gender issues were considered subjective and unscientific [25]. In open answers from a questionnaire to physician teachers about gender issues, many comments were minimising, neutralising, and sometimes even denying the role of gender in professional relationships [26].

In this paper, we theorise about and try to understand this resistance to a gender perspective in medicine in relation to what is considered good and valid research in the medical society.

## Methods

We used a theoretical model to explore and comprehend structures of scientific medicine and how they might influence the resistance to gender issues. The model was originally introduced by Johansson [27] to discuss pluralism and rationality of the social sciences. Johansson looks upon science as a social institution aiming at giving us a more 'truth-like' view of nature, man and society. He defines scientific rationality as the means to reach this 'truth-likeness'. The model was used to illustrate that a scientific field can embrace different kinds of research with different scientific rationalities.

**Figure 1:**
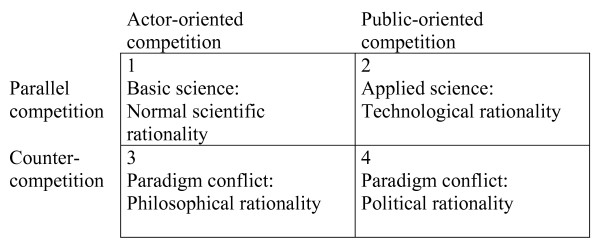
**Competition and rationality model modified from Johansson**. From: Johansson I. Pluralism and rationality in the social sciences. *Phil Soc Sci *1991, **21**(4):427–43.

One important presumption behind the theoretical model is that science as a social phenomenon is, like the rest of society, influenced by competition. For example, researchers compete for fame, resources and esteem. Four kinds of competition are described and related to each other in pairs. The first competition pair deals with the people to whom the research is addressed in the first place: to fellow researchers in the same field (actor-oriented) or to those who might benefit from the results of the research (public-oriented). The second competition pair deals with how the research is pursued: within (parallel competition) or outside (counter-competition) the prevailing paradigm. When social sciences were analysed through this competition model, Johansson distinguished four different research approaches, each with its own scientific rationality (Figure 1). In this paper, we have used the model to examine medicine.

## Results and discussion

**Figure 2:**
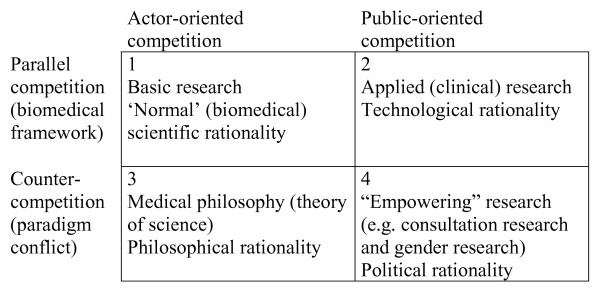
**Johansson's model applied to medicine: different types of research and research rationalities in medicine related to different types of competition**.

When applying the combinations in the model to medical science, we identified the following research areas in medicine (Figure 2): basic research, applied (clinical) research, medical philosophy and 'empowering' research.

### The biomedical framework versus other research paradigms

Basic and applied research is performed within the biomedical framework, the dominating paradigm in medicine. Researchers use the quantitative hypotheticodeductive method, with epistemological roots in positivism and logical empiricism. Knowledge is defined as facts that can be verified. Explanation and proof are sought. These traditional biomedical methods have been, and are, very successful in producing useful medical knowledge, but they are not suited for all types of medical research. For example, they are often not fitted to studies of 'soft' data such as patient experiences or patient-doctor interaction, which are important elements of clinical medicine. Nor can they help researchers to discover, interpret and understand the character and meaning of social phenomena, such as the reluctance to consider gender issues in medicine or to understand comprehensive processes in their contexts [28,29].

Medical researchers within medical philosophy and 'empowering' research recognise these limitations of the biomedical methods. They see paradoxes and medical anomalies, phenomena that do not comply with established biomedical explanations, for example, undefined musculoskeletal pain [30]. They adopt other research traditions, such as social constructivism, hermeneutics or phenomenology where knowledge is seen as interpreted, contextual and situated. The aim is to understand, not to prove. A paradigm conflict arises not only because of the epistemological difference from biomedicine, but also due to the character of the research subjects and because qualitative methods that are uncommon in the biomedical contexts are often used.

### Actor-oriented versus public-oriented research in medicine

Medical scientists within basic research and medical philosophy use a scientific language with a very specialised interdisciplinary terminology and a high theoretical abstraction in their research, which is often difficult to grasp for those who are not in the field. Thus, their primary scientific communication is with their fellow researchers in the same sector. In applied and 'empowering' research within medicine, on the other hand, researchers try to communicate with and address more directly those who might benefit from their scientific findings, as they are the ones that could evaluate the usefulness and efficacy of the research results.

### Scientific rationalities

In the original theoretical model (Figure 1), the scientific rationalities emerging from the analysis are classified as normal scientific, technological, philosophical, and political, respectively. Considering the divergent patterns regarding competition outlined above, we find that the scientific approaches of medicine and their rationalities can be described as follows (Figure 2):

i) Basic medical research. Most researchers in this sector do their research in pre-clinical departments of, for example, cellular morphology, biochemistry, physiology, molecular biology or immunology. In their laboratories, they conduct experiments and test hypotheses to gain new knowledge. The results seldom have immediate application in clinical practice. This research often uses the traditional 'orthodox' biomedical design, which has dominated modern medical science since the late 19th century, thus applying the 'normal' scientific rationality within medicine.

ii) Clinical (applied) research. A considerable part of medical research is within this field. It is carried out in clinical, public health and epidemiological departments, and in primary health care. It represents a wide range of research subjects, for example, the prevention and more efficient treatment of global health threats such as HIV/AIDS, tuberculosis, malaria and cancer, and in the prevention and treatment of 'welfare disorders', including adiposity, diabetes and hypertension. The research rationality is technological in a wide sense. It encompasses not only technical issues such as investigation methods or techniques to distribute medical technology and expertise, but it also includes various types of medical treatment and care.

iii) Medical philosophy and theory of science. This research area is not very widely represented in the core of medical science. The research deals with with the humanistic side of medicine: our choices, decisions and actions, critical self-reflection, and widening of perspectives. Researchers also problematise the definition, production and development of knowledge, and the handling of possible biases. Such research can be found in medical ethics, medical sociology and medical anthropology, and in gender-theory studies. The philosophical rationality implies the performance of analyses to elucidate different alternatives, not to provide distinct and given answers.

iv) 'Empowering' research. An important purpose of this research approach in medicine is to empower subordinated and oppressed groups or individuals as one way to ensure better health. For example, researchers within this area pay attention to the importance of position, living conditions and life experiences for health and disease. The power asymmetry between the patient and the caregiver is also focussed on. Much consultation research and research from a gender perspective is found here. The political scientific rationality holds action and change as significant constituents.

### Understanding the resistance to a gender perspective in medicine

In the original article on the theoretical model, Johansson concludes that a scientific community as a whole is rational when the different research approaches respect each other and when there is an interaction between the different sub-rationalities [31]. He states:

"It does not matter too much, then, if some scientists doing normal science are stubborn or dogmatic. Neither does it matter much if some theoreticians working with paradigm conflicts are highly speculative and totally insensitive to empirical findings. Nor does it matter if some scientists... merely think in terms of usefulness ... as long as there is an *interaction *between the different kinds of rationalities".

As we see it, medical science is not quite there yet. There is a clear hierarchy between different medical research fields. Basic research and, to some extent, traditional clinical research, are classified as genuine medical science. There is a special group of distinguished scientific journals with high impact, publishing preferably basic medical research. Lately, medical journals have also shown an increasing interest in clinical research where results can help combat global health problems in the developing as well as in the industrial world [31,32]. The other two research approaches, medical philosophy and 'empowering' research, are often considered as metaphysics or politics by authorities in medical academia, i.e. as something other than science.

Similarly, this has probably been the attitude encountered when trying to introduce into medicine the concept of gender as something more than biological sex. Attempts have ended up outside the prevailing paradigm, a low-status area, sometimes even ruled as non-scientific. The tradition of positivistic science seems to be a strong excuse for making medical research and medical education 'immune' to the gender discussion taking place in the academic world in other disciplines, and in society at large [24].

Representatives of biomedicine and those of a gender perspective not only compete for fame, esteem and resources; there is a wider conflict where biomedicine often claims the right to define the field and to preserve (gendered) privileges. To redefine gender as being synonymous with biological sex is one way to exert such rights. Unfortunately, such claims limit the scientific rationality of medicine. Biomedicine did not produce 'truth-likeness' for a series of important diseases until a gender perspective appeared and was used. We argue that a medical science with gender bias has a suboptimal scientific rationality.

### A wider application of the model

As exemplified in this paper, the theoretical model gave us a better understanding of the resistance to a gender perspective in clinical and academic medicine. This understanding has given us tools to use when handling gender issues in our work as faculty members and researchers.

We suggest that the model can be applied in a wider medical context when different research traditions collide and when misunderstandings between them arise, leading to the risk of restriction in the ways to gain 'truth-likeness' and thereby to a reduced scientific rationality. One example would be when there is scepticism about the scientific rigour of qualitative methods. Used in this way, the model could contribute to shape a medical community where pluralism is seen as a fruitful scientific challenge and benefit, not as a non-scientific disturbance or threat.

## Conclusion

• Research has revealed gender bias in many areas of clinical and academic medicine.

• To avoid such bias a gender perspective in medicine is needed.

• Resistance, difficulties and hard work have been reported from attempts to introduce gender issues into the medical world.

• The structures and hierarchies of medical science, where the biomedical framework dominates, can unfortunately contribute to negative attitudes to gender issues and a gender perspective in the medical society and thereby to gender bias.

• More scientific pluralism in medical science may help prevent gender bias in medicine.

## Competing interests

The author(s) declare that they have no competing interests.

## Authors' contributions

This article is an extension of a section in the thesis by GR *'I am solely a professional – neutral and genderless': On gender bias and gender awareness in the medical profession *(Umeå University 2004). KH and EJ were supervisors for GR while working on the thesis. All three authors contributed to the conception, design, and drafting of this article. GR was responsible for the final draft of the article.

## Pre-publication history

The pre-publication history for this paper can be accessed here:
